# Radiographic assessment of photodynamic therapy as an adjunctive treatment on induced periodontitis in immunosuppressed rats

**DOI:** 10.1590/S1678-77572010000300007

**Published:** 2010

**Authors:** Leandro Araújo FERNANDES, Thiago Marchi MARTINS, Juliano Milanezi de ALMEIDA, Letícia Helena THEODORO, Valdir Gouveia GARCIA

**Affiliations:** 1 DDS, MSc, Graduate student, Department of Surgery and Integrated Clinic, Araçatuba Dental School, São Paulo State University, Araçatuba, SP. Assistante Professor, Department of Pathology and Clinical Dentistry, Federal University of Piauí, PI, Brazil.; 2 DDS, MSc, PhD, Graduate student, Department of Surgery and Integrated Clinic, Araçatuba Dental School, São Paulo State University, Araçatuba, SP, Brazil.; 3 DDS, MSc, PhD, Associate Professor, Department of Periodontics, University Center of Educational Foundation of Barretos, Barretos Dental School, Barretos, SP, Brazil.; 4 DDS, MSc, PhD, Full Professor, Department of Surgery and Integrated Clinic, Araçatuba Dental School, São Paulo State University, Araçatuba, SP, Brazil.

**Keywords:** Periodontitis, Laser, Dexamethasone, Rats, Corticosteroids

## Abstract

**Objective:**

The aim of this study was to assess radiographically the effect of photodynamic therapy (PDT) as an adjunctive treatment to scaling and root planing (SRP) on induced periodontitis in dexamethasone-induced immunosuppressed rats.

**Material and Methods:**

The animals were divided into 2 groups: ND group (n=60): saline treatment; D group (n=60): dexamethasone treatment. In both ND and D groups, periodontal disease was induced by the placement of a ligature in the left first mandibular molar. After 7 days, ligature was removed and all animals received SRP, being divided according to the following treatments: SRP: saline and PDT: phenothiazinium dye (TBO) plus laser irradiation. Ten animals per treatment were killed at 7, 15 and 30 days. The distance between the cementoenamel junction and the height of the alveolar bone crest in the mesial surface of the mandibular left first molars was determined in millimeters in each radiograph. The radiographic values were analyzed statistically by ANOVA and Tukey's test at a p value <0.05.

**Results:**

Intragroup radiographic assessment (ND and D groups) showed that there was statistically significant less bone loss in the animals treated with PDT in all experimental periods compared to those submitted to SRP. Intergroup radiographic analysis (ND and D groups) demonstrated that there was greater bone loss in the ND group treated with SRP compared to the D group treated with PDT at 7 and 30 days.

**Conclusion:**

PDT was an effective adjunctive treatment to SRP on induced periodontitis in dexamethasone-induced immunosuppressed rats.

## INTRODUCTION

Periodontal disease is the result of the collapse of tooth supporting structures by the local action of periodontopathogenic microorganisms^24^. These microorganisms release substances that strictly injury periodontal tissues in addition to inducing tissue destruction by the host’s inflammatory and immunologic responses. Systemic factors such as diabetes, smoking, alcohol consumption^22^ and stress have been found to be associated with severe and/or rapidly progressive periodontitis. Furthermore, some medications have an impact on the periodontium and its response to bacterial plaque^20^.

In the last decades, organ transplant has become an accepted treatment for a range of acquired and congenital disorders. Corticoids are commonly used to treat many different diseases because of their anti-inflammatory effect and immunosuppressant properties. Glucocorticoids link to receptors inside the cell and cause redistribution of the lymphocytes. They also reduce T-cell proliferations, with a decrease in interleukin-2, and also downregulate interleukin-1 and interleukin-6, thereby attenuating the inflammation^26^.

Prolonged therapy with corticoids may favor osteoporosis, which is now regarded as a risk factor for periodontal disease^20^. The systemic use of drugs such as non-steroidal antiinflammatory substances and their possible effects on periodontal disease have been studied^6^. The use of corticoids can provoke from gingival ulceration up to downward migration of the epithelium, attachment loss and transeptal fiber disruption^6^. In addition, the systemic use of high doses of glucocorticoids leads to fibroblast activity inhibition, collagen and connective tissue loss, with decreasing rereepithelization and angiogenesis^14^ , reduction of number and activity of the osteoblasts, and increasing osteoclast function^18^. However, clinical studies are somewhat equivocal with respect to the effect of systemic glucocorticoids on periodontal tissues^13^.

Periodontal treatment is based on pathogenic microbiota reduction by scaling and root planing. However, mechanical therapy used alone can fail to eliminate pathogenic bacteria that are lodged deeply in the soft tissue, and also in inaccessible areas to the periodontal instruments, such as the furcation area and root depression^1^.

Systemic disease and adverse drug reactions address strategic challenges to the elaboration of a conventional periodontal treatment plan, leading to the use of complementary therapies in order to compensate for the intrinsic alterations related to periodontal repair process. Because of these limitations, adjuvant methods that provide for the elimination of periodontal pathogens have called the attention of many researchers, who consider antibiotic and antiseptic use as effective in the periodontal treatment^15^. On the other hand, there are also uncountable studies demonstrating the selection and resistance of bacteria provided by the overuse of antimicrobial drugs in the periodontal therapeutics^25,28^.

Recently, some *in vitro* ^7,17,30^ and *in vivo* ^2,4^ studies have shown satisfactory results with the use of photodynamic therapy (PDT). However, the introduction of PDT as an adjuvant periodontal treatment in immunosuppression conditions has not yet been reported in the literature.

This therapy consists in the association of a photosensitizer with an intense light source, both aiming to promote cellular death. The photodynamic activity of photosensitizers is based on photo-oxidative reactions that provide biochemical and morphological alterations in target cells. When the photosensitizer drug molecule absorbs light from a resonant energy, it is turned into a single exciting state. Depending on its molecular structure and environment, the molecule may then lose its energy by electronic or physical process, thus returning to the ground state, or it may undergo a transition to the triplet exciting state (unpaired electron spins). At this stage, the molecule may once more undergo electronic decay back to the ground state, it may either undergo redox reaction with its environment, or its excitatory energy may be transferred to molecular oxygen (also a molecular triplet-state) leading to the formation of the labile singlet oxygen (type-II reaction). This type of oxygen reactive species (ROS) is responsible for irreversible damage on bacterial cytoplasm membrane, including protein modification, respiratory chain breakdown and nucleic acid alterations^27^.

The major advantages of PDT are being a specific therapy for target cells, presenting no side effects, initiating its activity only when exposed to light, and supporting no resistant bacteria species selection^10^ , which is found to be rather common with the indiscriminate use of antibiotics^25^.

In this context, PDT may be an alternative adjuvant method for nonsurgical periodontal treatment under immunosuppressant conditions. Considering that prolonged use of corticoids is associated with the reduction of number and activity of the osteoblasts, and the increase of osteoclastic function^18^ , the aim of the present study was to compare the efficacy of PDT plus conventional mechanical therapy to scaling and root planing alone on alveolar bone loss in furcation areas of experimental periodontitis induced in rats either inhibited or not by dexamethasone.

## MATERIALS AND METHODS

This study was conducted on 120 adult male Wistar rats (120 to 140 g). The animals were kept in plastic cages with access to food and water *ad libitum.* Prior to the surgical procedures, all animals were allowed to acclimatize to the laboratory environment for a period of 5 days. All protocols described below were approved by the Institutional Review Board of Araçatuba Dental School, São Paulo State University, Araçatuba, SP, Brazil (Protocol no. 22/06).

### Drug administration protocol

Animals were then divided into 2 groups: D group (n=60), which received injections of 2 mg/kg body weight^15^ of dexamethasone (DeCADRON^®^ 2 mg, Prodome, Aché Pharmaceutical Laboratories SA, Campinas, SP, Brazil); and ND group (n=60), which received injections of 2 mg/kg body weight^14^ of saline. The subcutaneous injections were initiated 24 h before the experimental induction of periodontal disease and maintained every 3 days^6^ , during all the study period.

### Experimental periodontal disease protocol

General anesthesia was obtained by association of ketamine (0.4 mL/kg) and xylazine (0.2 mL/kg) via intramuscular injection. One mandibular left first molar of each animal in the ND and D groups was selected to receive a submarginal cotton ligature in order to induce experimental periodontitis^12,19^. After 7 days of periodontal disease induction, the ligature was removed from all animals of both groups. The left molars were then submitted to scaling and root planning (SRP) with Mini Five 1314 curettes (Hu-Friedy Co. Inc., Chicago, IL, USA) through 10 distal-mesial traction movements in both buccal and lingual aspects of the teeth. The furcation and interproximal areas were scaled with the same curettes through cervical-occlusal traction movements. Scaling and root planing was performed by the same experienced operator. The animals of each group (ND and D) were randomly assigned to one of the two treatments proposed (30 animals/treatment): SRP: the mandibular left molars were submitted to SRP and irrigation with 1 mL of saline; and PDT: the mandibular left molars were submitted to SRP and irrigation with 1 mL of phenothiazinium dye (TBO - Toluidine Blue-O; Sigma Chemical Co., St. Louis, MO, USA) (100 µg/ mL) solution, followed by application of a low-level laser (LLL) source. Saline and TBO were slowly poured into the periodontal pocket with a syringe (1 mL) and an insulin needle (13 mm x 0.45 mm) (Becton Dickinson Ind. Ltd, Curitiba, PR, Brazil) without bevel.

The LLL source used in this study was galliumaluminum-arsenide (GaAlAs) (GaAlAs; Laser Bio Wave LLLT; Kondortech equipment, São Carlos, SP, Brazil) with wavelength of 660 nm and spot size of 0.07 cm^2^. After 1 min of TBO application, the LLL was applied in 3 equidistant points at each buccal and lingual aspect of the first mandibular molar in contact with the tissue. The laser was delivered during 133 s *per* point, with power of 0.03 W, power density of 0.428 W/cm^2^ and energy of 4 J/ point (57.14 J/cm^2^/point). The area received a total energy of 24 J.

### Experimental periods

Ten animals of each group and treatment were killed at 7, 15 and 30 days after the periodontal disease treatment by administration of a lethal dose of thiopental (150 mg/kg) (Cristália Ltd, Itapira, SP, Brazil). The jaws were removed and fixed in 10% neutral formalin for 48 h.

### Radiographic analysis

Rat left hemi-mandibles were removed to determine the level of bone loss. Standardized radiographs were obtained with the use of digital radiographic images provided by the Digora computerized imaging system (Soredex, Orion Corporation, Helsinki, Finland), which uses a sensor instead of an x-ray film. Electronic sensors were exposed to 70 kV and 8 mA with exposure time of 0.4 seconds. The source-to-film distance was 50 cm. The distance between the cementum-enamel junction and the height of alveolar bone was determined for the mesial root surface of mandibular left first molars^2^. Millimeters of bone loss for each radiograph were measured three times in a blind fashion by the same examiner.

### Intraexaminer reproducibility

Before the radiographic analysis was performed, the examiner was trained by double measurements of 20 specimens, with a 1-week interval. Paired t-test statistics was run and no differences were observed in the mean values for comparison (p value = 0.51). Additionally, Pearson's correlation coefficient was obtained between the 2 measurements and revealed a very high correlation (0.99, p = 0.000).

### Statistical Analysis

The hypothesis that there were no differences in bone loss rate in the furcation region between treatment groups was tested using the Bioestat 3.0 software (Bioestat, Windows 1995, Sonopress Brazilian Industry, Manaus, AM, Brazil).

After the normality of radiographic data was analyzed by Shapiro-Wilk test, the intragroup and intergroup analysis was carried out with a two-way ANOVA followed by Tukey's test. A significance level of 5% was set for all analysis.

## RESULTS

### Clinical analysis

All non-dexamethasone animals (ND Group), regardless of the treatment, presented no clinical differences in general health, and showed weight gain within the predicted range for healthy rats (Table 1). All dexamethasone-treated animals (D Group) presented progressive weight loss in a significant level when compared to those in the ND group (Table 1), which show trends of immunosuppression and systemic alterations.

**Table 1:** Mean ± Standard Deviation (SD) of body weight (g) in each group, treatment and period

**ND group- non-dexamethasone (saline)**				
**Groups**				
**Periods**	**Initial periods**	**7 days**	**15 days**	**30 days**
**Treatments**				
SRP (n=30)	245.85 ± 4.18 *	262.28 ± 2.05 * ^&^ †	282.85 ± 1.46 * ^&^ †	306.00 ± 0.81 * ^&^ †
PDT (n=30)	247.28 ± 5.31 *	261.42 ± 1.61 * ^&^ †	284.14 ± 2.03 * ^&^ †	307.85 ± 1.95 * ^&^ †
N	60	20	20	20
**D group - dexamethasone**				
**Groups**				
**Periods **	**Initial periods**	**7 days**	**15 days **	**30 days**
**Treatments**				
SRP (n=30)	246.85 ± 5.6 *	218.00 ± 1.29 * ^&^ †	198.28 ± 1.49 * ^&^ †	177.14 ± 1.34 * ^&^ †
PDT (n=30)	246.57 ± 4.92 *	219.14 ± 1.21 * ^&^ †	199.14 ± 2.19 * ^&^ †	178.28 ± 1.11 * ^&^ †
N	60	20	20	20

* Significant difference among experimental periods (Initial, 7, 15, and 30 days) in the same group and treatment (p<0.05). ANOVA and Tukey’s test. & Significant difference between groups in the same treatment and period (p<0.05). ANOVA and Tukey’s test.

† Significant difference between different groups and treatments in the same period (p<0.05). Tukey’s test. SRP= Scaling and Root Planning; PDT= Photodynamic Therapy

### Radiographic analysis

**Figure 1:**
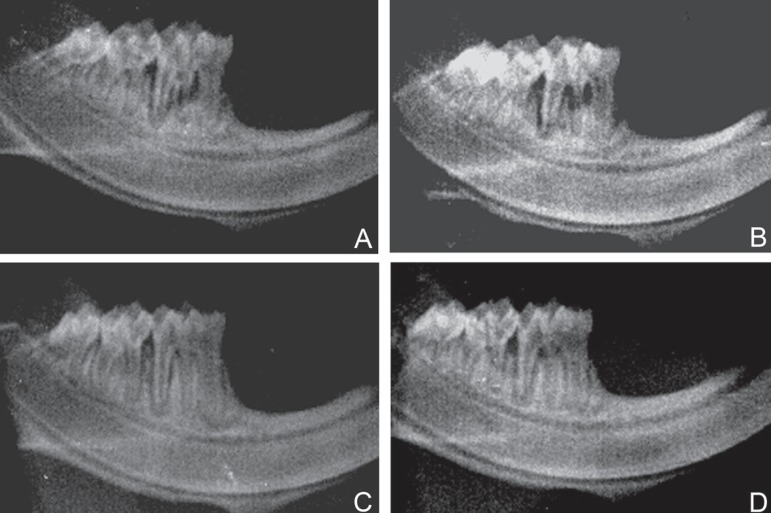
Bone loss area in the mesial region of mandibular first molar: (A) ND group/SRP/30 days; (B) D group/SRP/30 days; (C) group ND/PDT/30 days; (D) D group/PDT/30 days. *SRP= Scaling and Root Planning; PDT= Photodynamic Therapy*

Intragroup radiographic assessment (ND and D) showed that there was significantly less bone loss in the animals treated with PDT in all experimental periods than in those treated with SRP (Figure 1, Table 2). Intergroup radiographic analysis (ND and D groups) demonstrated greater bone loss in the ND group treated with SRP compared that the D group treated with PDT, at both 7- and 30-day periods (Figure 1, Table 2).

**Table 2:** Mean ± Standard Deviation (SD) of the distance between the cementoenamel junction and the alveolar bone crest (mm) on the mesial surface of the mandibular first molars in each group, treatment and period

**ND- non-dexamethasone (saline)**			
**Groups**			
**Periods**	7 days	15 days	30 days
**Treatments**			
SRP	1.12 ± 0.05 * ^&^ †	1.06 ± 0.03 * ^&^	1.03 ± 0.07 * ^&^ †
PDT	0.80 ± 0.10 †	0.73 ± 0.03 †	0.75 ± 0.07 †
N	20	20	20
**D - dexamethasone**			
**Groups**			
**Periods**	**7 days**	**15 days**	**30 days**
**Treatments**			
SRP	1.40 ± 0.16 * ^&^ †	1.49 ± 0.29 * ^&^ †	1.50 ± 0.15 * ^&^ †
PDT	0.90 ± 0.02 †	0.87 ± 0.09	0.82 ± 0.06 †
N	20	20	20

* Significant difference with SRP + PDT treatment in the same period and group (p<0.05). ANOVA and Tukey’s test. & Significant difference between groups in the same treatment and period (p<0.05). ANOVA and Tukey’s test.

† Significant difference and between different groups and treatment in the same period (p<0.05). ANOVA and Tukey’s test. SRP= Scaling and Root Planning; PDT= Photodynamic Therapy

## DISCUSSION

This study compared the influence of PDT as an adjuvant treatment on induced periodontitis in dexamethasone-induced immunosuppressed rats. In the present study, the induced periodontal disease was characterized by clinical signs of gingival inflammation, such as edema, redness and attachment loss of tooth gingival tissue. In the dexamethasone-inhibited animals (D group), the clinical signs of gingival inflammation were more exacerbated, characterized as: a greater bone loss in the furcation region, connective tissue disorganization, discreet fibroblasts and intense inflammatory infiltrate in all experimental periods, when compared to non-inhibited rats (ND).

The animals treated with this drug presented lethargy, hematoma and alopecia at the moment of sacrifice. Furthermore, there was a significant weight reduction throughout the present study. This fact probably occurred because the drug decreases gastrointestinal nutrient absorption^11^. These alterations have already been reported^9^ , showing a trend towards immunosuppression and systemic alterations.

The results of the present study have also demonstrated that the animals in the D group presented a greater bone loss in the furcation area, as well as more disorganized connective tissue when compared to the animals in the ND group. These alterations were described in another study that has also evaluated the corticoid effects upon periodontal tissues^6^.

On the other hand, a clinical study has not demonstrated influence of corticosteroid therapy on clinical parameters of periodontal disease in patients suffering from neurological disease^13^. The use of high doses of corticoid leads to a reduction of number and activity of osteoblasts, and an increase in the osteoclastic functions^18^. It also reduces gastrointestinal calcium absorption, which, in turn, results in lower blood calcium levels, and triggers PTH secretion that leads to systemic bone resorption^23^. However, another clinical study on liver transplant recipient has demonstrated that the doses of glucocorticoids have not influenced alveolar bone loss, although there was an inverse relationship with the duration of treatment^13^.

Corticoids can lead to healing process delay by decreasing angiogenesis and capillary proliferation, which reduces blood flow^14^. They also interfere in phagocytosis and antigen digestion, inhibiting macrophage migrations and stabilizing lysosomes, avoiding proteolytic enzymes release. In addition, they modify fibroblast functions, delaying their migration, damaging type-I and type-II pro-collagen synthesis by modifying mRNA and mitotic activity^17^.

The number of studies investigating the PDT antimicrobial effects has increased. This therapy consists of the association of a photosensitizing agent with a light source, being initially used for oncology treatment^27^. Studies have shown favorable results using PDT principles against microorganisms involved in periodontitis^29^ and periimplantitis^21^.

The radiographic findings showed that the animals of the ND and D groups that received PDT treatment presented less significant bone loss than those treated with SRP alone, in all experimental periods. These results are in accordance with the literature, which has demonstrated PDT effectiveness in periodontal treatment for both animals^2^ and humans^4^.

The beneficial effect of PDT as an adjuvant method to conventional mechanical treatment of periodontal disease, both in dexamethasone-inhibited and non-inhibited rats, was probably caused by the photo-destructive effects on the different ROS, mediated by type-I reaction (initiated by superoxide, anionic hydroxyl or free radicals) or by type-II reaction (initiated by singlet oxygen). These oxygen-reactive species are responsible for irreversible damage on bacterial cytoplasmic membrane, including protein modification, respiratory chain breakdown and nucleic acid alterations^27^.

It was also evident in the present study that the animals in the D group that received PDT presented less bone loss when compared to those in the ND group that received SRP treatment alone, at both 7- and 30-day periods. The beneficial effects of PDT in the periodontal disease could be explained not only by the local antimicrobial activity, previously described, but also by the increasing angiogenesis that brings more oxygenation to the area^5^.

Another possible explanation for the results obtained could be the biomodulation action of the low-intensity laser alone. Studies have reported that the use of this source accelerates bone repair, presents antiinflammatory effect, favors the cellular chemotaxis^8^ , and promotes local vasodilatation and angiogenesis^25^. Thus, it could increase oxygen diffusion through the tissue, favoring the repair process because collagen secretion by fibroblasts in the extracellular space occurs only in the presence of high rates of oxygen pressure^16^.

Systemic corticoid use has been indicated in low and high doses for many treatments such as mucocutaneous and respiratory diseases, tendinitis, bursitis, arthritis and cysts in general^3^ ; it is also used in all levels of immunotherapy, based on the need and regimen prescribed by the individual practitioner^26^. One of the side effects of this drug is the increasing infection risk because of the inhibition effects of cellular immunity, which could cause more severe periodontal damages^6^ , as demonstrated in this study.

Considering these facts, the application of alternative or adjuvant periodontal therapies to SRP conventional treatment, such as the use of systemic antibiotics, has been indicated, in spite of the disadvantage in developing bacterial drug resistance^25,28^. In this context, the use of local bactericidal agents would aid the periodontitis treatment.

The conventional periodontal treatment presents local limitations, such as effectiveness of mechanical instrumentation in difficult access areas, e.g., furcation region. PDT is not affected by this limitation as it is based on a photosensitizer agent associated with light emission, such as laser irradiation. Other advantages of PDT is having no side effect, initiating its activity only when exposed to a light source, and preventing from supporting resistant bacteria species selection^9^.

## CONCLUSIONS

Within the limitations of this study, it may be concluded that PDT was effective as a SRP adjuvant treatment for bone loss reduction in induced experimental periodontitis when compared to conventional nonsurgical treatment, both in normal rats and in systemic dexamethasone-inhibited animals.
